# Low HbA1c With Normal Hemoglobin in a Diabetes Patient Caused by *PIEZO1* Gene Variant: A Case Report

**DOI:** 10.3389/fendo.2020.00356

**Published:** 2020-06-19

**Authors:** An Song, Lin Lu, Yuxiu Li, Mei Lin, Xingxing Yuan, Xinqi Cheng, Weibo Xia, Ou Wang, Xiaoping Xing

**Affiliations:** ^1^Key Laboratory of Endocrinology of National Health Commission, Department of Endocrinology, Peking Union Medical College Hospital, Chinese Academy of Medical Science and Peking Union Medical College, Beijing, China; ^2^Department of Internal Medicine, Peking Union Medical College Hospital, Peking Union Medical College, Chinese Academy of Medical Sciences, Beijing, China; ^3^Department of Laboratory, Peking Union Medical College Hospital, Peking Union Medical College, Chinese Academy of Medical Sciences, Beijing, China

**Keywords:** diabetes, glycated hemoglobin, glycated albumin, hemolytic anemia, *PIEZO1* gene

## Abstract

**Background:** Diabetes is a global disease with rapidly increasing prevalence in the world. Glycated hemoglobin (HbA1c) as an important indicator of diabetes could reflect the average serum glucose level over 120 days. However, when using HbA1c to diagnose diabetes, it is important to consider other factors that may impact HbA1c level including age, race/ethnicity, detection method, and co-morbidities. Here we report a case of diabetes with normal hemoglobin but reduced HbA1c.

**Case report:** A 57-year-old female patient was diagnosed with diabetes by oral glucose tolerance test results. However, the HbA1c level was repeatedly decreased, glycated albumin level was high, with normal levels of hemoglobin and albumin, and a slightly elevated level of bilirubin. Moreover, life span of red blood cells was significantly shortened. Further examination of whole exome sequencing of the patient and her daughter showed heterozygous variant in *PIEZO1* gene (c.6017T > A) in both, which is associated with dehydration hereditary stomatocytosis (DHS). After this diagnosis, we changed nateglinide to sitagliptin to reduce the burden of the pancreas islet function.

**Conclusion:** In case of abnormally low HbA1c, we recommend that GA and reticulocyte should be measured simultaneously. Moreover, the methodology for hemoglobin measurement and the diseases that could cause abnormal quantity and quality of red blood cells and hemoglobin be considered.

## Introduction

Diabetes is a global disease with rapidly increasing prevalence in the world. The American Diabetes Association has recommended glycated hemoglobin (HbA1c) as an important indicator of diabetes and a useful monitoring tool for long-term glycemic control (1). The reference range of HbA1c derived from DCCT/UKPDS is 4–6%. Since the life span of red blood cells in circulation is 120 days, HbA1c percentage reflects the average serum glucose level over 120 days, with biological variation < 2% (2). However, when using HbA1c to diagnose diabetes, it is important to consider other factors that may impact HbA1c level including age, race/ethnicity, detection method, and co-morbidities (1). While diabetes is indicated by high levels of HbA1c, there are also probable causes that are indicated by abnormally low HbA1c levels (< 4%). The possible causes include laboratory error, extreme diet control, excessive use of antidiabetic drugs, and anemia/hemoglobinopathies (3). Here we report a case of diabetes with normal hemoglobin but reduced HbA1c. Hemolytic diseases with normal hemoglobin are not uncommon. However, their influence on the determination of glycosylated hemoglobin is still poorly understood.

## Case Presentation

In 2010, a 57-year-old female patient presented with mild polydipsia, polyuria, blurred vision, and weight loss. Diabetes mellitus was diagnosed by oral glucose tolerance test results (OGTT, determination of glucose and insulin at 0 and 120 min). The HbA1c level at that time was 3.6% and glycated albumin (GA) was 16.3% (normal range: 10.8–17.1%). Type 1 diabetes-associated antibodies such as Islet Cell Antibodies (ICA), Glutamic Acid Decarboxylase antibodies (GAD), Insulin Autoantibodies (IAA), and insulinoma-antigen 2 (IA2A) were all negative. Total bilirubin (TBil) was 38.7 umol/L (normal range: 5.1–22.2) and direct bilirubin (DBil) was 11.6 umol/L (normal range: 0–6.8). Other laboratory tests including serum alanine transaminase (ALT), albumin, and renal function test were all within the normal range. Upon diagnosing her with diabetes mellitus, the primary doctor prescribed nateglinide to control the hyperglycemia. In recent years, her HbA1c level were found repeatedly reduced, while the level of GA remained high. Hemoglobin (Hgb) and albumin were still in the normal range, and the bilirubin level was found slightly elevated. In terms of screening for diabetic complications, the patient had completed the fundus examination, renal ultrasound, cardiac ultrasound, lower limb artery ultrasound, 24 h urine protein, and urine albumin-to-creatinine ratio, and no abnormalities were found. However, the carotid ultrasound showed the presence of atherosclerotic plaques. Past medical history showed the patient suffered from keratoconjunctivitis sicca for 10 years and carotid atherosclerosis for 3 years. The family history showed her mother also suffered from diabetes mellitus with similarly low HbA1c level (3.4%) and slightly decreased Hgb (102 G/L). Her mother also had Sjogren syndrome and passed away at age of 70 years old without further investigation of the causes of low HbA1c. In addition, both her mother and daughter had hyperbilirubinemia. The complete blood count of the daughter showed that normal Hgb (156 G/L) and increased reticulocyte (169.9 × 10^9^/L, normal range: 24.0–84.0 × 10^9^/L), normal white blood cell (6.55 × 10^9^/L) and Platelet (321 × 10^9^/L).

**Figure 1:**
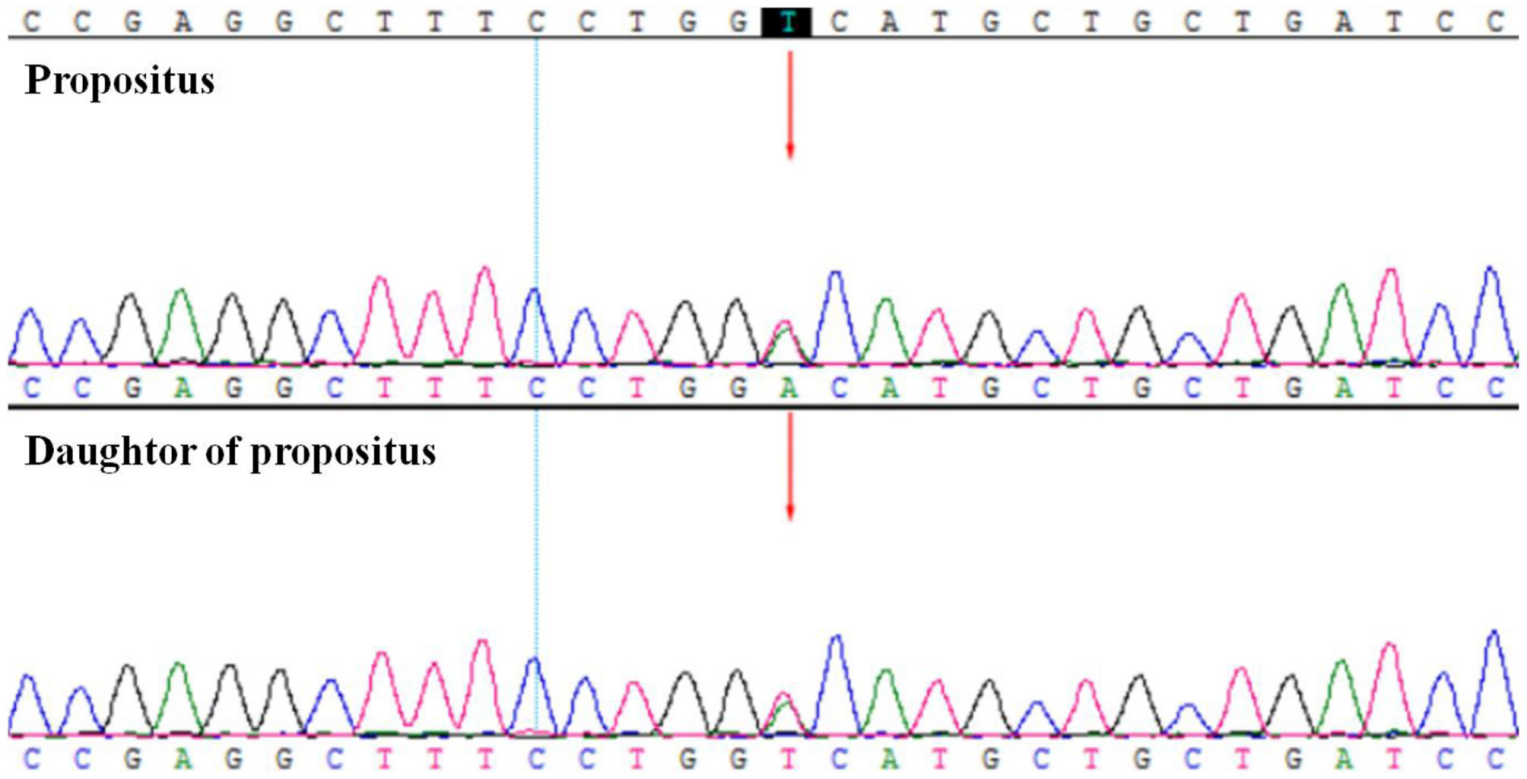
The whole exome sequencing and Sanger sequencing verification showed the patient (propositus) and her daughter both had the heterozygous variant of *PIEZO1*; c.6017T>A; p.V2006D.

After recent hospitalization in our medical center, the abnormally low HbA1c levels attracted our attention. To confirm the problem, we performed repeated HbA1c test by both ion-exchange chromatography and immunization method. However, the low HbA1c level persisted. Hemoglobin electrophoresis revealed no abnormality. After excluding laboratory error and variation of hemoglobin, we performed further laboratory examinations (Table 1): Hgb was 129 G/L, reticulocyte was 265.4 × 10^9^/L (normal range: 24.0–84.0 × 10^9^/L), TBil was 29 umol/L, DBil was 7.6 umol/L, and the life span of red blood cells measured by CO breath test was significantly shortened to 43 days (normal range: > 75 days). All these results above indicated the presence of hemolytic anemia. However, screening for common causes (erythrocyte osmotic fragility, 6-phosphate glucose dehydrogenase test and plasma free hemoglobin test, Ham test, Rous test, and Coombs test were all in normal range) of acquired hemolytic disease led to exclusion of autoimmune hemolysis, glucose-6-phosphate dehydrogenase deficiency, paroxysmal hemoglobinuria, and so on. Pancreatic MRI showed slightly atrophied pancreatic body, but iron deposition in the pancreas was normal (23.7 ms at T2 ^*^). Both the patient and her daughter showed heterozygous variant in *PIEZO1* gene (c.6017T > A, p.V2006D) by whole exome sequencing of the blood with confirmation of sanger sequencing (Figure 1), which is associated with dehydration hereditary stomatocytosis (DHS). After this diagnosis, nateglinide was changed to sitagliptin to reduce the burden of the pancrea islet function.

**Table 1:** Clinical features and laboratory results of this patient.

		**Clinical features and laboratory findings**		**Reference range**	
Age (year)		57			
Weight (kg)		50			
Height (cm)		159			
BMI (kg/m^2^)		19.78			
HbA1c (%)		3.4		4.5–6.3	
GA (%)		14.5		10.8–17.1	
Hgb (g/L)		129		120–160	
Free hemoglobin (mg/dl)		2.3		0–5	
Reticulocyte (×109/L)		265.40		24.0–84.0	
Reticulocyte (%)		7.49		0.8–2	
MCV (fl)		102.8		82.0–97.0	
MCH (pg)		36.6		27.0–32.0	
MCHC (g/L)		356		320–360	
RDW (%)		14.6		0.0–15.0	
Hematocrit (%)		36.4		35.0–50.0	
Platelet (×10^9^/L)		191		100–350	
WBC (×10^9^/L)		5.59		3.5–9.5	
Iron (μg/dl)		253.8		50–170	
Ferritin (ng/ml)		269		14–307	
TIBC (μg/dl)		256		250–450	
TS (%)		91.8		25.0–50.0	
ALT(U/L)		25		9–50	
ALB(g/L)		43		35–52	
TBIL(μmol/L)		29		5.1–22.2	
DBIL(μmol/L)		7.6		0.0–6.8	
LDH (U/L)		177		0–250	
K (mmol/L)		4.9		3.5–5.5	
**OGTT**	**0 h**	**0.5 h**	**1 h**	**2 h**	**3 h**
GLU (mmol/L)	5.5	8.0	13.1	13.1	10.6
INS (μIU/mL)	4.51	17.66	40.88	41.77	41.65
C-P (ng/mL)	0.93	1.83	3.51	5.65	6.15

*BMI, body mass index; underweight ≤ 18.5, normal weight = 18.5–24.9, overweight = 25–29.9, obesity = BMI of 30 or greater; HbA1c, glycated hemoglobin; GA, glycated albumin; Hgb, hemoglobin; MCV, mean corpuscular volume; MCH, mean corpuscular hemoglobin; MCHC, mean corpuscular hemoglobin concentration; RDW, red cell distribution width; WBC, white blood cell; TIBC, total iron binding capacity; TS, transferrin saturation; ALT, alanine transaminase; ALB, albumin; TBIL, total bilirubin; DBIL, direct bilirubin; LDH, lactate dehydrogenase; K, serum potassium; OGTT, oral glucose tolerance test; GLU, glucose; INS, insulin; C-P, C-Peptide*.

## Discussion

In the absence of other confounding factors, HbA1c correlates with mean blood glucose (MBG) and GA to some extent. There were some studies about the correlation of HbA1c with MBG. In a study by Nathan et al., a total of 268 patients with type 1 diabetes, 159 patients with type 2 diabetes, and 80 non-diabetic subjects were enrolled (4). Continuous glucose monitoring (CGM) and self-monitoring of blood glucose (SMBG) were recorded for 3 months and was used to calculate their MBG. For total participants, MBG = 1.59 × HbAlc-2.59, while for the diabetes patients, MBG = 1.57 × HbAlc-2.44 (*R*^2^ = 0.79, *P* < 0.01). The corresponding relationship between HbAlc and MBG obtained from this study is given in *2019 standards of medical care in diabetes* by the American Diabetes Association (ADA) (Table 2) (1). However, the limitation of this study was that it mostly incorporated non-Hispanic whites, and therefore may not have been reflective of findings in the general population. In a similar study by Ma et al., from 2007 to 2010, 318 patients were investigated, including 115 cases of the normal control group, 57 cases of impaired glucose regulation group, and 146 cases of newly diagnosed type 2 diabetes mellitus group. This study primarily investigated the relationship between MBG and HbAlc in the Chinese population (Table 2) (5). For the total participants, MBG = 1.252 × HbAlc-0.992 (*R*^2^ = 0.718, *P* < 0.01); for newly diagnosed T2DM patients, MBG = 1.255 × HbAlc-0.886 (*R*^2^ = 0.621, *P* < 0.01). These studies demonstrated that the relationships between HbA1c and MBG hold irrespective of the demographics being studied, and therefore this finding has clinical significance.

**Table 2:** Mean glucose levels for specified HbA1c and GA levels.

**HbA1c (%)**	**Non-Hispanic whites (1)**	**Chinese population** **(****5**, **6****)**	
	**MBG (mmol/l, 95%CI)**	**MBG (mmol/l,95%CI)**	**GA (%,95%CI)**
5.0		5.3 (4.3–6.3)	14.2 (12.9–15.5)
5.5		5.9 (4.8–7.0)	–
6.0	7.0 (5.5–8.5)	6.5 (5.4–7.6)	17.1 (15.7–18.5)
6.5		7.1 (6.0–8.3)	19.6 (17.0–22.1)
7.0	8.6 (6.8–10.3)	7.8 (6.6–9.0)	20.0 (18.5–21.5)
7.5		8.4 (7.2–9.6)	21.4 (19.8–23.0)
8.0	10.2 (8.1–12.1)	9.0 (7.7–10.3)	22.9 (21.2–24.5)
8.5		9.7 (8.3–11.0)	–
9.0	11.8 (9.4–13.9)	10.3 (8.9–11.6)	25.7 (24.0–27.5)
9.5		10.9 (9.5–12.3)	-
10.0	13.4 (10.7–15.7)	11.5 (10.1–13.0)	28.6 (26.7–30.5)
10.5		12.2 (10.7–13.7)	–
11	14.9 (12.0–17.5)	12.8 (11.2–14.3)	31.5 (29.5–33.5)
12	16.5 (13.3–19.3)	14.0 (12.4–15.7)	34.3 (32.2–36.4)

*HbA1c, Glycated hemoglobin; MBG, Mean blood glucose: GA, Glycated albumin*.

For the correlation between HbA1c and GA, in another study, Ma et al. collected the HbA1c and GA level of 2,532 patients (898 in the normal control group, 695 in impaired glucose regulation group, and 939 in newly diagnosed T2DM group), and found that there was a significant positive correlation (*R* = 0.701, *P* < 0.01) between the two factors (6). The regression equation obtained was GA = 2.871 × HbA1c-0.112; that means when HbA1c increased by 1%, GA increased by 2.87%.

Based on the above correlation of HbA1c with MBG and with GA, we determined the levels of MBG and GA in the present case. Here, the patient was a middle-aged woman with a chronic disease course and a clear diagnosis of diabetes mellitus. According to hospital findings, her HbA1c was <4% (Hgb and Alb levels were normal), and GA was more than 14.5%. However, upon considering the relationship between HbA1c with MBG and with GA, and using the above formula, the patient's MBG was determined to be 3.26 mmol/L and GA to be 9.65%. It could be seen that the GA and actual glucose levels did not match the level of HbA1c; hence, we established the diagnosis of low glycosylated hemoglobin in this patient.

When HbA1c does not match the actual blood glucose situation, factors that may interfere with HbA1c should be firstly considered (1, 7). Methodology-specific interference factors included: (1) Abnormal HbA1c measurements in hemoglobinopathy patients due to variation in hemoglobin levels and the type of measurement method. (2) Pseudoelevation of HbA1c measurement in patients with nephropathy, which may occur due to the formulation of hemoglobin. (3) HbA1c levels detected by ion-exchange chromatography, which can be affected by the charge status of glycosylated and non-glycosylated hemoglobin and can be interfered with by the intermediate (Schiff base) of the HbA1c formation process. All of the above interference factors can be determined by changing the detection methodologies and paying attention to whether any hemoglobin variant is found in the detection process.

In this case, the hemoglobin was detected by ion-exchange chromatography in our hospital, without hemoglobin variant detection in the map and hemoglobin abnormality findings by hemoglobin electrophoresis. The immunoassay was also done and the level of HbA1c was still detected to be low (3.6%), which suggested that interference with HbA1c due to detection methodology could be excluded. Other diseases that could cause low HbA1c level include hemolytic diseases, such as erythrocyte membranopathies, immunohemolytic anemia, hemoglobinopathy, and erythrocyte enzymopathy (8), insulinoma, drugs, pregnancy, rapidly progressing type 1 diabetes, severe jaundice, hyperlipidemia, and high doses of vitamins C and vitamins E (9). The patient denied any history of hypoglycemia, anemia, and large doses of vitamin intake. After combining the patient's family history of low HbA1c with the laboratory examinations of elevated reticulocyte level (7.49%), elevated indirect bilirubin level, and shortened erythrocyte life span (43 days), we first considered the repeated reduction in HbA1c to be associated with hereditary hemolytic disease. However, after preliminary examination, the patient ruled out the common cause of hereditary hemolysis. Then we performed genetic sequencing and detected a heterozygous variant in *PIEZO1* gene. The mutations of this gene may be associated with dehydration hereditary stomatocytosis (DHS) (10, 11), which is also known as hereditary xerocytosis. DHS is a rare congenital hemolytic anemia with prevalence estimates of 1: 50 000 (12). It is usually compensatory (moderate anemia, high reticulocyte count) (13). However, with long-term disease development, high hemolytic complications (biliary stones and iron overload) may occur, leading to its aggravation. The *PIEZO1* gene encodes for a large transmembrane PIEZO1 cation channel (14, 15). Electrophysiological studies have demonstrated that mutations in the *PIEZO1* gene might cause delayed inactivation of ion channels, thereby increasing cation permeability, leading to dehydration of red blood cells in patients with DHS (16). Most DHS-associated *PIEZO1* mutations are in the highly conserved COOH-terminal region, such as p.R2456H, p.T2127M, and p.E2496ELE, which have been identified in more than 50% of DHS cases (17). The specific site of this new variant is c.6017T>A (p.V2006D), which is also in the COOH-terminal region with an extremely low frequency in the population. There is no relevant literature report on the pathogenicity of this variant at present, and the variant could be classified as uncertain clinical significance (VUS) according to the ACMG Standards and Guidelines. In addition, protein function prediction tools such as Sorting Intolerant From Tolerant (SIFT), Mutation Taster, and Polymorphism Phenotyping version 2 (Polyphen-2) indicated that it could be a pathogenic mutation. The score of SIFT is 0, Polyphen2 HumVar score is 0.834, and the prediction of Mutation Taster is disease-causing. All the evidences indicated the high possibility of the pathogenic genetic mutation and protein changes.

It is believed that hereditary hemolysis is also related to the onset of diabetes mellitus. However, the mechanism is not clear. Current hypotheses regarding the mechanism include insulin resistance resulting from iron overload induced by hemolysis, or autoimmune response to pancreatic beta cells resulting from pancreatic beta-cell apoptosis, and inflammation of parenchymal organs including the pancreas (18–21). The additional causes could be the restriction of adequate insulin secretion due to zinc deficiency from anemia (22). For this patient, the decrease in fasting and post-prandial insulin level was not consistent with typical T2DM, suggesting that there may be a specific relationship between DHS and the onset of diabetes mellitus. Recent studies indicated that PIEZO1 is a mechanically activated ion channel that might mediate pressure-induced pancreatitis and regulate diet-induced systemic insulin resistance (23, 24). However, there was no evidence proving the mutation on the *PIEZO1* gene could directly affect the onset of diabetes. Russo et al. investigated seventy-four hereditary anemias patients by the targeted-NGS panel and found that the coinheritance of *PIEZO1* and *SEC23B* causative mutations resulted in marked iron overload, with very high ferritin levels (1938 ng/mL) and increased transferrin saturation (TSAT 88%) (25). Moreover, Orvain et al. investigated 4 DHS patients and indicated that severe iron overload is frequent in DHS patients despite well-compensated hemolysis and no or little transfusion requirement (26). Recently, Andolfo I et al. demonstrated that functional characterization of erythroferrone (ERFE)-A260S variant could directly caused hepatic iron overload by impairing the BMP-SMAD pathway in the congenital dyserythropoietic anemia type II cases with biallelic mutations of SEC23B gene (27). However, more studies on the molecular mechanisms of the dysregulation of iron homeostasis in PIEZO1 gain-of-function mutations were required in the future.

Additionally, when serum glucose was not consistent with HbA1c, GA should be measured simultaneously since it is unaffected in patients with hemolysis (28). There has been a significantly negative correlation between the GA/HbA1c ratio and hemoglobin in patients with hemolysis (*R* = −0.710), including diabetic patients, so that the GA/HbA1c ratio could reflect the degree of hemolysis in diabetic patients with hemolytic anemia (29).

In the case of abnormally low HbA1c with normal Hgb, we recommend that the methodology for hemoglobin measurement and the diseases that could cause abnormal quantity and quality of red blood cells and hemoglobin be considered. Since hemolysis with normal Hgb is not uncommon, the clinical significance of reticulocyte level should not be ignored. At the same time, it should be noted that hereditary hemolysis may have the relationship with diabetes mellitus, and therefore, we suggest that attention be given to reducing the burden of the pancreas when oral drugs are used to control serum glucose.

## Data Availability Statement

The original contributions presented in the study are included in the article/supplementary material, further inquiries can be directed to the corresponding author/s.

## Ethics Statement

The studies involving human participants were reviewed and approved by Peking union meidical college hospital. The patients/participants provided their written informed consent to participate in this study. Written informed consent was obtained from the individual(s) for the publication of any potentially identifiable images or data included in this articles.

## Author Contributions

We declare that AS, YL, ML, XY, and XC contributed to the analysis of the results and to the writing of the manuscript. LL, WX, OW, and XX contributed to read, and approved the final, submitted version of the manuscript. All authors contributed to the article and approved the submitted version.

## Conflict of Interest

The authors declare that the research was conducted in the absence of any commercial or financial relationships that could be construed as a potential conflict of interest.
